# Attitudes and Beliefs of Wild Boar Hunters in Croatia Towards Preventing and Controlling African Swine Fever

**DOI:** 10.3390/ani15192782

**Published:** 2025-09-24

**Authors:** Lucija Pečurlić, Tihomir Florijančić, Neška Vukšić Končevski, Denis Deže, Sanja Jelić Milković

**Affiliations:** 1 Faculty of Agrobiotechnical Sciences Osijek, Josip Juraj Strossmayer University of Osijek, Vladimira Preloga 1, 31000 Osijek, Croatia; lucija.bencaric@fazos.hr (L.P.); tihomir.florijancic@fazos.hr (T.F.); denis.deze@fazos.hr (D.D.); 2 Croatian Hunting Federation, Vladimira Nazora 63, 10000 Zagreb, Croatia; neska.vuksic@hls.com.hr

**Keywords:** African swine fever, wild boar, wild boar hunters’ attitudes, disease prevention, biosecurity measures, Croatia, wildlife management, education and awareness

## Abstract

African swine fever (ASF) is a highly contagious and fatal disease of domestic pigs and wild boars, with severe economic and ecological consequences. Wild boar hunters play a critical role in the early detection and control of ASF due to their direct interaction with wild boar populations. This study examined the attitudes, knowledge, and behaviour of wild boar hunters in Croatia regarding ASF prevention and control, with a focus on the influence of sociodemographic factors, hunting experience, and participation in training programmes. An online survey of 276 wild boar hunters from an ASF-affected county in Croatia was conducted between October and December 2024. Results indicate that 93.5% of wild boar hunters are aware of ASF and its risks, relying primarily on internet sources for information. Experienced and higher-educated wild boar hunters demonstrated greater confidence in recognising ASF symptoms and stronger support for preventive measures, education, and institutional cooperation. Factor analysis revealed three main dimensions shaping attitudes: communication and awareness, institutional capacity, and regulatory policies. The results emphasise the importance of continuous education, transparent communication and participatory approaches to strengthen cooperation with hunters and improve ASF control. They also emphasise the need for targeted, evidence-based communication strategies that actively involve hunters in surveillance and reporting. Tailored educational materials and digital communication could increase carcass reporting, improve early detection and increase the overall effectiveness of ASF control programmes.

## Simple Summary

African swine fever is a deadly disease that affects pigs and wild boars, causing major economic losses and threatening food production. Since there is no vaccine, the prevention and early detection of the disease depend on strict safety measures and the cooperation of key groups, including wild boar hunters. This study explored the knowledge, attitudes, and behaviour of wild boar hunters in Croatia regarding the prevention and control of this disease. We conducted an online survey among wild boar hunters to assess their experience, awareness, and willingness to engage in training and reporting. The results show that 93.5% of wild boar hunters are well informed about the disease, mainly through the internet, and 72.8% are concerned about its spread. Experienced wild boar hunters and those with higher education are more confident in recognising sick animals and support preventive measures such as testing, fencing, and selective hunting. However, attendance at educational seminars is still limited. The study highlights that wild boar hunters strongly support better education, cooperation with authorities, and government investment in control measures. These findings are valuable for improving communication strategies and building trust between wild boar hunters and institutions, which is crucial for preventing the spread of this serious animal disease.

## 1. Introduction

### 1.1. History and Spread of African Swine Fever (ASF)

African swine fever (ASF) is a highly contagious viral disease affecting both domestic pigs and wild boar, causing severe economic losses and threatening animal health and food security across Europe and globally. The disease is caused by African swine fever virus (ASFV), a DNA virus of the *Asfarviridae* family, which spreads through direct contact, contaminated materials, and human-mediated transport of infected products [1,2]. Due to its high mortality rate and the absence of effective vaccines or treatments, ASF control relies heavily on strict biosafety measures, rapid detection, and population management [1,3,4].

African swine fever (ASF) was first reported in the early 20th century in Kenya, from where it gradually spread to other parts of Africa [1]. During the second half of the 20th century, the disease spread beyond Africa, first to the Iberian Peninsula, where it caused major epizootics in Spain and Portugal [5]. Since 2007, ASF has re-entered Europe, appearing in Georgia and subsequently spreading through Eastern Europe, Russia, and the Baltic states, such as Lithuania and Latvia, where the first cases were recorded in 2014 [6,7,8]. This epidemic predominantly involved wild boar, serving as the ecological reservoir that sustained virus circulation and facilitated further geographic spread [9,10]. The spread within the European Union is linked to wild boar migration and human factors, including improper disposal of contaminated food waste [3]. Rogoll et al. [11] report that African swine fever (ASF) has affected numerous European Union member states, including Estonia, Latvia, Lithuania, Poland, Germany, Czechia, Slovakia, Hungary, Romania, Bulgaria, Belgium, and Italy, with confirmed cases in both wild boar populations and domestic pig farms. In addition to Europe, ASF was also reported in China in 2018, leading to the mass depopulation of pigs in the country, which is the world’s largest pork producer [1]. Croatia did not remain unscathed either. ASF was first confirmed in Croatia in 2023 in domestic pigs on two small outdoor farms, and by the end of the same year, a total of 1124 cases had been recorded in domestic pigs and 11 in wild boars [12]. In response to the epidemic, various control measures were implemented. In Croatia, the control of African swine fever (ASF) in wild boar populations is based on a combination of hunting regulations, carcass monitoring and disposal, and movement restrictions. Wild boar populations are controlled through intensified, regulated hunting with strict biosecurity requirements for hunters and hunting grounds [13]. An important measure is passive surveillance, where hunters and landowners are obliged to report sick or dead wild boars, which must then be sampled and safely disposed of via authorised Category 1 processing plants to reduce the persistence of the virus in the environment [13,14]. In addition, movement restrictions and zoning are applied under the EU ASF regionalisation legislation (Commission Implementing Regulation (EU) 2023/594 [15], implemented in Croatia by Narodne novine [13]), which prohibits the movement of live wild boar and restricts activities within restricted, protection, and surveillance zones. These measures, which are coordinated by the national ASF surveillance programme, form the legal and practical framework that directly influences the activities of hunters and their attitude towards ASF prevention and control. During 2025, ASF was confirmed in a total of 21 wild boars and 11 pig farms in Croatia [16].

### 1.2. Hunting Practices and Hunter Demographics in Croatia

Hunters in Croatia are individuals who have passed the hunting exam and carry out essential tasks of game management, including breeding, protection, hunting, and the sustainable use of wildlife, in line with established customs and ethics. The hunting year runs from 1 April to 31 March, and all activities are regulated by the Act on Hunting under the supervision of the Ministry of Agriculture, with data maintained in the Central Hunting Register [17]. Hunters are typically members of local associations and county federations, which are coordinated nationally by the Croatian Hunting Association [17,18].

As of 2023, Croatia had 68,292 registered hunters, representing about 1.8% of the population. This number has been increasing in recent years (65,402 in 2022; 64,245 in 2021) [18]. Game harvest statistics also illustrate the dynamics of wildlife populations: between 2022 and 2023, wild boar numbers rose slightly (from ~49,000 to ~50,000), while roe deer and red deer declined marginally. Among small game, hare numbers increased, common pheasant decreased, and waterfowl remained stable [18]. These figures reflect the balance and fluctuations typical of Croatian hunting.

The demographic profile of Croatian hunters shows a strong predominance of men, who make up more than 95% of the membership of hunting associations. The age structure indicates a prevalence of middle-aged and older adults: the largest group of hunters is between 45 and 65 years old, with an average age of 49 years. Women remain significantly underrepresented, although their membership has been gradually increasing in recent years [18]. According to the study by Pejnović et al. [19] and Tolušić [20], the majority of hunters have completed secondary education (more than 60%), while a smaller proportion hold higher or university degrees. Most hunters are either employed or retired, and they predominantly live in rural or semi-rural areas, which aligns with the spatial characteristics of hunting grounds. The sociodemographic structure of hunters is also linked to patterns of participation in educational activities, seminars, and preventive programs, with younger hunters tending to seek information online and via social media, while older hunters prefer traditional methods of knowledge transfer. Given the ageing and predominantly male hunter population in Croatia, understanding how age, education and gender influence ASF risk perception and prevention behaviour is crucial for designing effective outreach.

### 1.3. The Role of Wild Boar and Wild Boar Hunters in African Swine Fever (ASF) Control

Wild boars are a critical reservoir for African swine fever (ASF), significantly contributing to virus persistence in the environment [1,21]. Wild boar hunters, with their direct contact with wild boar, routine surveillance practices, and role in carcass detection and removal, are therefore essential in early warning systems and control [7,22,23]. Their knowledge, behaviour, and readiness to cooperate significantly influence the success of ASF control efforts [6,22]. Studies across Europe have consistently shown variability in wild boar hunters’ awareness, motivations, and compliance with ASF-related measures [6,7,22]. Although many wild boar hunters acknowledge the seriousness of ASF, communication with authorities is often perceived as inadequate, and some recommended practices are viewed as impractical or detached from ground realities [6,7]. Participation increases significantly when wild boar hunters are involved in planning and implementation of measures, and when mutual trust with institutions exists [7,22]. Participatory approaches such as workshops, focus groups, and collaborative mapping have proven effective in enhancing cooperation and contextual understanding [22]. Wild boar hunters often interpret ASF not merely as a veterinary concern but as one impacting their cultural identity and community practices, especially when decisions are made without their input [22,24]. Biosafety education and awareness campaigns tailored to wild boar hunters have shown to improve compliance, though information channels are frequently reported as fragmented or misaligned with wild boar hunters’ needs [5,6]. ASF epidemics have been shown to alter wild boar population dynamics and behaviour, complicating surveillance and control, with disease-induced mortality often exceeding hunting-induced mortality [25,26]. Environmental factors such as habitat fragmentation and hunting pressure further exacerbate virus transmission risks [26]. Integrated management strategies combining carcass removal, population control, and enhanced biosafety have been shown to significantly slow ASF spread, but rely on the active engagement of trained wild boar hunters implementing protocols correctly [1,2,21]. Coordination between wild boar hunters and domestic pig farmers is also essential, as differing perceptions and practices between these stakeholder groups can complicate disease management. Successful ASF control requires a unified, multi-stakeholder approach [5].

Although attitudes towards ASF prevention have been analysed in several European countries, there are no comparable results from Croatia. Little is known about how Croatian hunters perceive their role in ASF control, including their trust in veterinary authorities, possible obstacles to reporting carcasses and their willingness to take biosecurity measures such as testing or selective hunting. Clarifying these unknowns is crucial for the development of communication strategies and participatory approaches that ensure cooperation with wild boar hunters and improve the effectiveness of national ASF control programmes. This represents a significant research gap, especially given the confirmation of ASF outbreaks in domestic pigs and wild boar in Croatia since 2023. Addressing this gap is important to tailor communication strategies, training programmes and policies to the specific socio-demographic and cultural context of Croatian hunters. Given the reemergence of ASF in Croatia in 2025 and the implementation of general control measures that also include hunting practices, the need to examine the attitudes, knowledge and awareness of wild boar hunters as key stakeholders in the early detection and control of the spread of the disease was recognized. The main objective of this study is therefore to investigate the attitudes, knowledge, awareness, and behaviour of wild boar hunters in relation to African swine fever (ASF), with a particular focus on the relationship between hunting experience, education, and training, while identifying the key factors that shape hunters’ perceptions of preventive measures, educational initiatives, cooperation with institutions, and support for regulatory measures to combat ASF. Following this, the study addresses the following research questions:

1. What are the sociodemographic characteristics of wild boar hunters (age, gender, education, and experience), and how do these factors influence their hunting activity and participation in educational programs?
2. To what extent are wild boar hunters informed about African swine fever, and what are their primary sources of information?
3. Is there an association between hunting experience and wild boar hunters’ awareness, behavior, and confidence in recognizing ASF symptoms?
4. Which preventive and control measures against ASF do wild boar hunters most strongly support, and how do they perceive the importance of education, cooperation, and institutional capacity in combating ASF?
5. Which underlying factors (ASF communication, education and awareness; institutional capacity and coordination; regulation and disease control policy) can be identified through factor analysis of wild boar hunters’ attitudes, and how do these factors vary with age, education, training participation, and concern about ASF?
  - How do hunters perceive the effectiveness of current ASF communication and control measures?
  - What are hunters’ attitudes towards potential future ASF management strategies?
6. How do sociodemographic characteristics and participation in training programs influence wild boar hunters’ attitudes toward regulatory measures, preventive actions, and institutional support for ASF control?

## 2. Materials and Methods

The primary data were collected using the survey method, whereby an online questionnaire was used as a research instrument. The survey was conducted online using the web-based application Microsoft Forms (Microsoft Corporation), as it offers advantages over face-to-face, telephone, or postal data collection, such as covering a wider geographical area, quick response time, lower costs and fewer errors [27,28,29,30]. However, there are also some disadvantages of using online surveys, including the availability of computers and Internet access, digital literacy, sampling and representativeness issues, and non-response bias, age and education of respondents [28,30]. These factors may affect the representativeness of the sample and thus limit the generalizability of the findings, particularly with regard to older or less digitally active hunters.

Given that obtaining a probability sample accurately representing the population was not feasible, a non-probability sample was used. The survey was distributed between October and December 2024 via multiple channels, including emails to the Croatian Hunting Federation, hunters’ associations, and social media ensuring broad coverage across different hunting groups. In addition, a post hoc analysis of the response time or page timing was conducted to exclude a possible bias in the sample in this study. According to Vecchio et al. [31], those who had a completion and response time of less than five minutes were removed from the sample because they were inattentive during the online survey, meaning that the respondents completed the survey too quickly or answered the questions randomly. In accordance with the law on the protection of personal data and the guarantee of digital rights, all participants were informed before the start of the survey about the study procedure and data management, as well as that their participation is voluntary and that they therefore have the right to withdraw from the study at any time and without giving any reason.

The target group of respondents were professional and recreational wild boar hunters from Osijek-Baranja County. This county was chosen because it is one of the centres of intensive pig production in Croatia, has one of the largest hunting communities and extensive hunting areas, and was one of the first regions affected by African swine fever after neighbouring Vukovar-Srijem County. Our final sample of 276 respondents represented about 9.9% of the total of 2782 registered hunters in Osijek-Baranja County, organised in 99 hunting clubs [32]. A total, of 336 questionnaires were collected. Of these, 24 responses were excluded due to implausibly short completion times, and 36 respondents withdrew before completing the questionnaire, resulting in a final analytical sample of 276 respondents. The questionnaire consisted of 39 open and closed questions divided into several sections: socio-demographic characteristics, hunting experience and frequency, and knowledge of African swine fever. Further sections dealt with hunters’ attitudes towards preventive measures, education and awareness, cooperation and support, as well as limited resources and the need for investment, which were rated on a 5-point Likert scale (1 = strongly disagree to 5 = strongly agree). These statements were selected based on a review of relevant literature [33,34,35,36,37,38,39]. The statistical analysis was carried out using the statistical software package SPSS Statistics, version 26.0 (IBM Corp., Armonk, NY, USA) [40]. The collected data were analysed in three different steps. The data were analysed using descriptive statistics (frequency analysis, arithmetic mean, mode, median and standard deviations), non-parametric test chi-square test (χ^2^) and parametric test (independent t-test and ANOVA) and multivariate analysis (factor analysis). A descriptive statistical analysis was used to describe the socio-demographic characteristics of the sample, wild boar hunters’ experience and activity, hunting frequency, hunters’ sense of security in recognising symptoms of African swine fever in wild boars. The chi-square test (χ^2^) was performed to determine the significant associations between the questions on wild boar hunters’ experience and activity, hunting frequency and hunters’ sense of security in recognising the symptoms of African swine fever (ASF). Factor analysis was conducted with the aim of reducing the number of variables into a smaller set of components (factors) relating to statements about wild boar hunters’ opinions on prevention measures, education and awareness, cooperation and support, and limited resources and the need for investment. Factor analysis identified underlying themes in hunter attitudes, grouping correlated survey items into interpretable factors to reduce complexity. To examine the suitability of each intercorrelation matrix for factor analysis, we performed the Kaiser-Meyer-Olkin measure of sampling adequacy (KMO) and Bartlett’s test of sphericity. The extraction method used in the factor analysis was principal component analysis, and we used factors with an eigenvalue greater than 1. Varimax rotation was performed to facilitate the interpretation of each factor. To check the reliability of the factor analysis, Cronbach’s alphas were calculated. Independent t-test and ANOVA were performed to determine the significant differences between the factor scores and the questions.

The results were illustrated in suggestive graphics and tables and have been interpreted accordingly.

## 3. Results

### 3.1. Sample Description

The sample consisted of 93.8% male respondents, with the largest proportion (47.5%) aged between 35 and 54 and the smallest proportion (8.0%) aged 55 or older. Two thirds (67.0%) had a secondary education, while 33.0% had higher education. About half (51.1%) had more than five years of hunting experience, and most hunted recreationally (92.8%). Individual hunting (55.4%) was more common than group hunting (44.6%). Most hunters were members of associations (81.5%), although 58.7% rarely attended seminars (Table 1).

In the survey, we also asked hunters how much they knew about African swine fever (ASF). A total of 93.5% stated that they are informed about African swine fever, and most (66.3%) had heard about it on the Internet (web portals for hunters and social networks), while other sources of information are television, radio, newspapers (26.1%) and from the Ministry of Agriculture, Forestry and Fisheries and the Croatian Hunting Federation (7.6%). Most were concerned about the spread (72.8%) and more than half (55.8%) felt confident in recognising infected animals. We also asked the hunters surveyed to choose more than one option regarding the measures that should be taken to prevent the spread of the disease. They chose zoning and movement restrictions, polymerase chain reaction (PCR) testing of dead wild boars, PCR testing of shot wild boars, installation of physical barriers (fences), depopulation (removal of wild boar populations from affected areas to prevent further spread of ASF), selective hunting and vaccination of wild boars. Almost all respondents (96.0%) were of the opinion that vaccination should be government-funded if it were available in the future (Table 1).

### 3.2. Hunting Experience as a Predictor of Behaviour, Awareness, and Attitudes Toward ASF

**Figure 1:**
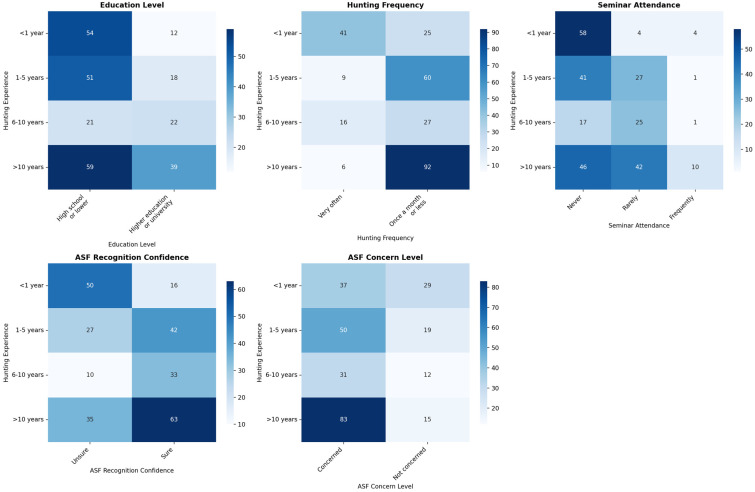
Association strength between hunting experience and ASF knowledge/attitudes/behaviours (χ^2^).

Associations between hunters’ experience and level of education, hunting frequency, participation in training, confidence in ASF detection and concern about the spread of ASF were determined using the chi-square test (χ^2^). The results of the chi-square test (χ^2^) are shown in Figure 1.

Hunters with six or more years of experience were more likely to have a higher level of education than less experienced hunters (χ^2^ = 16.516, df = 3, *p* = 0.001, Cramer’s V = 0.245). For example, 51.2% of hunters with six to ten years of experience and 39.8% of those with more than ten years of experience had a university degree, compared to only 18.2% of those with less than one year of experience.

There was a strong and highly significant relationship between experience and hunting frequency (χ^2^ = 73.551, df = 3, *p* < 0.001, Cramer’s V = 0.516). Hunters with more experience reported hunting more frequently throughout the year: 93.9% of hunters with more than ten years of experience hunted frequently, compared to only 37.9% of hunters with less than one year of experience.

Experience was also significantly associated with participation in hunting seminars or training. (χ^2^ = 45.082, df = 6, *p* < 0.001, Cramer’s V = 0.286). Less experienced hunters overwhelmingly reported never or rarely participating, while those with more than six years of experience reported participating more frequently in such events (although overall participation remained modest).

Hunters with longer experience were significantly more aware of ASF symptoms/transmission routes than less experienced hunters (χ^2^ = 37.877, df = 3, *p* < 0.001, Cramer’s V = 0.370). A majority of hunters with more than five years of experience considered themselves confident in recognising ASF, compared to only 24.2% of those with less than one year of experience. Hunting experience was significantly associated with the level of concern about ASF (χ^2^ = 16.365, df = 3, *p* = 0.001, Cramer’s V = 0.244). Concern increased with experience: while 56.1% of respondents with less than one year of experience expressed concern, this proportion increased to 84.7% among respondents with more than ten years of experience.

### 3.3. Wild Boar Hunters’ Perception of Preventive Measures, Education and Cooperation in the Fight Against African Swine Fever (ASF)

To address research question 4, hunters were asked to indicate their level of agreement with a series of statements regarding preventive and control measures against ASF, as well as the importance of education, cooperation, and institutional capacity. Table 2 presents the mean scores and standard deviations for these statements, which also served as the basis for the factor analysis in research question 5. Table 2 shows the degree of hunters’ agreement (measured on a Likert scale from 1 to 5) with various statements on combating ASF, education, cooperation and prevention. In addition to the mean (M) and standard deviation (SD), the median (Me), the mode (Mo), and the percentage of respondents who agreed or strongly agreed with the statements were given for each.

Overall, the responses showed a strong consensus in favour of education and awareness and cooperation and support statements. The highest levels of approval were for supporting hunters in the fight against ASF (92.4%) and encouraging hunters to cooperate and share information on suspected ASF cases (90.2%). Cooperation between hunters and collaboration with local and international authorities was also favoured by over 84.1% of respondents. The statements on education and awareness-raising also met with great approval. Between 82.6% and 87.7% of hunters agreed that the proper disposal of wild boar remains, the regular updating of information on ASF, and the education of the hunters and local population are important.

In contrast, approval for preventive and regulatory measures was comparatively lower. While increased monitoring of wild boar (76.8%), general preventive measures (67.1%) and stricter border controls (66.7%) were still viewed favourably, only around half of respondents (53.6%) agreed with the need for stricter regulatory measures, indicating less consensus on more restrictive measures.

### 3.4. Factor Analysis of Hunters’ Views on African Swine Fever: Prevention, Education, and Cooperation

Factor analysis was performed to determine a smaller number of factors that represent the interrelationship among nineteen statements regarding preventive measures, education and awareness about ASF, collaboration and support and limited resources and investments in infrastructure for ASF control and prevention (Table 3). Principal component analysis was used considering eigenvalues greater than 1. Varimax rotation with Kaiser normalization was applied to improve the interpretation of factor scores obtained by factor analysis. In order to confirm that data are suitable for analysis, the Kaiser–Mayer–Olkin (KMO) measure of sampling adequacy was applied and gave a value of 0.945, which is great and Bartlett’s test of sphericity (χ^2^ = 5153.191, *p* < 0.01) also confirmed that data could be used in factor analysis. The factor analysis revealed three factors: ASF communication, education, and awareness (F1), institutional capacity and coordination (F2), and regulation and disease control policy (F3), which together explained 75.0% of the total variance. Calculated Cronbach’s Alphas coefficients verified the consistency and the reliability of principal component analysis (0.966 to 0.834).

According to the data in Table 3, the first factor explained 34.92% of the variance. Considering the variable most strongly associated with the first factor, it was called ASF communication, education, and awareness, and this component accounts for the greatest variability of the variance explained. The second factor explained 24.05% of the variance and groups the variables related to the hunters’ support for stronger institutional involvement, intersectoral cooperation, and investment in ASF control infrastructure so this factor was called institutional capacity and coordination. The third factor (regulation and disease control policy) explained 16.02% of the variance. The third factor reflects hunters’ support for legal regulations, surveillance, border control, and preventive actions to limit the spread of ASF.

### 3.5. Connection of Sociodemographic Characteristics of Respondents, Participation in Training, Concern About the Spread of ASF Regarding Factor Scores

Differences in the average values of factor scores between respondents’ sociodemographic characteristics, participations in training and concerns about the spread of ASF, was examined using an ANOVA and independent *t*-test.

**Figure 2:**
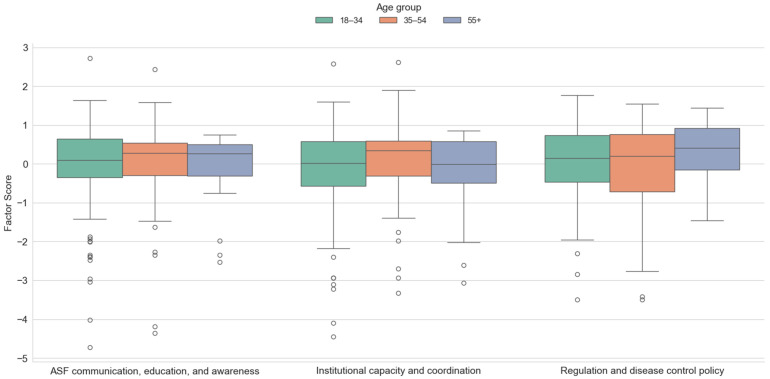
Differences between investigated factors scores with regard to the age of respondents.

The results presented in Figure 2 show significant differences between the age group of the respondents and the factor values for the variables institutional capacity and coordination (F = 3.146, *p* < 0.05). The highest mean score for the factor value institutional capacity and coordination was found for hunters aged 35 to 54 years (M = 0.15, SD = 0.853), while younger hunters aged 18 to 34 years orientated themselves more towards the variables of factors regulation and disease control. In order to determine between which groups there is a statistically significant difference in the average values of the factor scores, multiple comparisons of the arithmetic mean values were carried out using the LSD post hoc test (least significant difference). The results of the LSD test show that only the difference in the mean values of the factor determined in the case of the second factor (institutional capacity and coordination) and the group of youngest hunters (18–34) and the middle age group of hunters aged 35 to 54 is significant, while all other combinations are not statistically significant. Boxplots show a small number of respondents with very low factor scores across all three dimensions, indicating a group of hunters with markedly lower trust in institutions, weaker engagement with ASF communication, and lower support for regulatory measures. These outliers may represent hunters with negative past experiences with veterinary authorities or opposition to government interventions.

**Figure 3:**
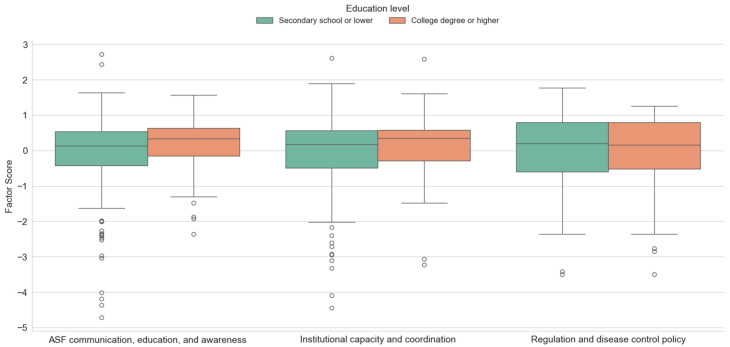
Differences between investigated factors scores with regard to the education of respondents.

The results of an independent samples t-test, shown in Figure 3, show that higher education group shows a slightly higher median and narrower interquartile range. Results indicate that there is a significant difference between the ASF communication, education and awareness factor (*t* = −2.642, *p* < 0.01). The highest mean factor score in the case of the first factor was obtained for hunters with higher education, which means that hunters with higher education pay more attention to the variables expressed by the factor ASF communication, education and awareness (M = 0.19, SD = 0.708), while for hunters with secondary or lower education the variables of this factor are not important (M = −0.09, SD = 1.105). Other factor values were not found to be statistically significant in relation to hunter education. However, according to the arithmetic mean, institutional capacity and coordination are important for hunters with higher education, while the variables under the factor regulation and disease control are important for hunters with secondary education. The presence of individual outliers in the boxplots reflects the natural variability of respondents’ opinions. These outliers suggest that while most hunters follow a similar pattern, a few show markedly different perceptions, possibly due to personal experience, greater knowledge or varying levels of information about ASF.

**Figure 4:**
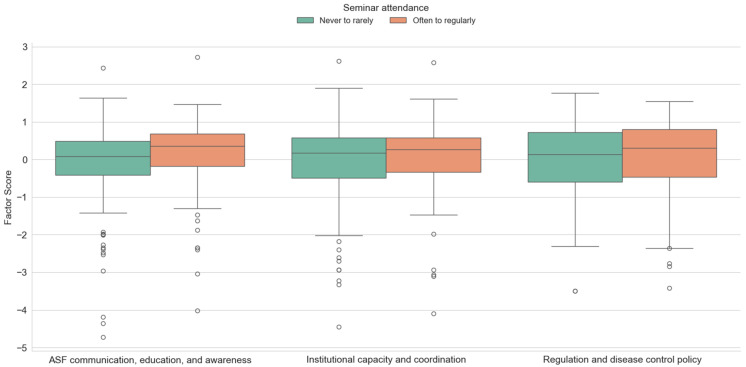
Differences between investigated factors scores with regard to the seminar attendance.

The results of a *t*-test for independent samples, shown in Figure 4, indicate that there is a significant difference between the ASF factor communication, education and awareness (*t* = −2.251, *p* < 0.05). The highest mean factor score in the case of the first factor was obtained for hunters who regularly participate in hunter seminars and training (M = 0.16, SD = 0.925), while for hunters who rarely participate in seminars and training, variables of this factor are not important (M = −0.11, SD = 1.038). Other factor values proved to be statistically insignificant in relation to hunter education. However, according to the arithmetic mean, institutional capacity and coordination as well as regulation and disease control policy are important for hunters who attend seminars and training courses for hunters more regularly. The concentration of negative outliers among hunters who rarely attend seminars may indicate a subgroup that is disengaged from formal training opportunities and holds more negative perceptions of ASF communication, education and awareness and institutional capacity and coordination.

**Figure 5:**
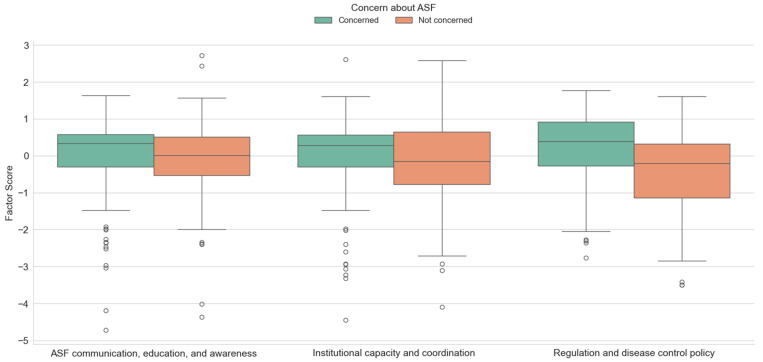
Differences between investigated factors scores with regard to the hunters concerns about ASF.

The results of a *t*-test for independent samples, shown in Figure 5, indicate that there is a significant difference between the third factor (regulation and disease control policy) (t = 4.060, *p* < 0.01). Respondents who are concerned about ASF have slightly higher median and upper quartile values, suggesting more positive attitudes toward regulation and control measures. The highest mean factor score in the case of the third factor was obtained for hunters concerned about the spread of ASF among wild boars and domestic pigs (M = 0.16, SD = 0.935), while for hunters who are not concerned, the variables of this factor are not important (M = −0.43, SD = 1.145). Other factor values were not found to be statistically significant in relation to hunters’ concern about the spread of ASF. However, according to the arithmetic mean, communication, education and awareness as well as institutional capacity and coordination are important for hunters concerned about the spread of ASF. Several extreme low-scoring outliers were observed among hunters who reported not being concerned about ASF, particularly for the factors related to ASF communication, education and awareness, and regulation and disease control policy. These individuals may represent a subgroup with very negative attitudes toward veterinary authorities or government-led disease control measures, possibly reflecting distrust or opposition to such interventions.

## 4. Discussion

The majority of items assessing attitudes toward ASF prevention and control received high average scores (above 4 on a 5-point Likert scale), indicating strong support among hunters for education, cooperation, reporting, and preventive actions. While this is a positive outcome, it may also point to a potential ceiling effect, where the clustering of responses at the upper end of the scale reduces variability and limits the ability to detect subtle differences between respondent groups. Additionally, the possibility of social desirability bias cannot be excluded, as participants may have been inclined to express agreement with socially approved statements. These factors should be taken into account when interpreting the results. Future studies should consider combining self-report surveys with complementary methods, such as behavioural observation (e.g., monitoring reporting rates or hunting practises), indirect questioning techniques to reduce social pressure, or choice experiments in which respondents are forced to make trade-offs between competing ASF control measures. In addition, increasing the sample size and ensuring greater diversity in terms of age, education, and digital literacy would improve representativeness and reduce the influence of sampling bias.

The structure of the sample in this study is broadly comparable to trends observed in European hunting populations, where males, older age groups and hunters with secondary education or lower educational qualifications predominate in recreational hunting. A similar demographic pattern is evident in Germany and Lithuania, where women remain significantly underrepresented in hunting, while hunters with higher education show a greater tendency toward training and keeping up to date with current information on wildlife diseases [6,24,41,42,43]. Our results reflect these trends and align with the demographic characteristics previously described by Pejnović et al. [19] for Croatian hunters, confirming that hunting remains a predominantly male and middle-aged activity, despite a slight increase in female participation compared to historical data.

The high level of awareness about African swine fever (ASF) among Croatian hunters is consistent with findings from Latvia and Estonia, where hunters actively follow information on diseases that may affect wild boar populations [7,22]. This awareness is characterised by a generational shift in information channels, as our respondents predominantly (66.3%) rely on online platforms, as opposed to traditional media, as emphasised in previous studies such as Pejnović et al. [19]. As the majority of hunters stated that they receive information about ASF mainly through internet portals and social media, official communication strategies should therefore be adapted to these favoured channels. The development of targeted digital materials such as infographics, short educational videos, and evidence-based explanations could help to align online discourse more closely with official guidelines and improve the consistency and credibility of messages and also tailoring communications to less-educated hunters using non-technical language could improve information uptake. However, studies from Germany emphasize that declarative awareness does not necessarily imply accurate recognition of disease symptoms, making continuous education essential for reducing misjudgements in the field [24]. This concern is relevant for our findings as well, since formal participation in seminars and training remains limited, echoing observations by Pejnović et al. [19] regarding insufficient engagement in structured educational activities among Croatian hunters. Analyses conducted in the Baltic countries also showed that more experienced hunters are more likely to recognize the importance of formal education, while younger hunters often rely more on information from social media and online portals [22,44]. Previous research shows that hunters play a crucial role in the early detection of ASF and the implementation of biosecurity measures [3]. Latvian and Estonian hunters demonstrated a willingness to cooperate, and report suspected cases, particularly when there is clear communication and coordination with the competent authorities [7,22]. This highlights the importance of institutional support and transparent information sharing, which has also been recognized in this study. In many European countries, including Lithuania, the lack of continuous contact between hunting associations and veterinary services is cited as a major obstacle to effective disease control [6,44]. European Food Safety Authority (EFSA) emphasizes that the combination of local training and online materials can ensure greater outreach and a better understanding of biosecurity protocols [3]. Although participation in training remains limited, our results show that experienced hunters regularly attend seminars and also score significantly higher on the ASF communication, education and awareness factor. This highlights the need for structured and motivating training programmes that combine face-to-face workshops with online modules to improve accessibility.

The factor analysis of hunters’ attitudes in this study highlights three key areas: communication and awareness-raising, institutional coordination, and regulatory measures. Similar dimensions have been identified in Latvia, where hunters emphasized that an effective fight against ASF is inconceivable without clear legal frameworks as well as systematic education on game handling and proper disposal of remains [7]. In Germany, the need for better coordination between state institutions, local hunting associations, and international organizations has been emphasized, particularly in border areas [24]. Previous experiences in Lithuania and Poland show that hunters who have better cooperation with institutions also demonstrate a higher level of acceptance of preventive measures, including depopulation and the installation of physical barriers [6,25]. However, depopulation remains a controversial measure that sparks debates within both the professional and hunting communities, as highlighted by Dixon et al. [1], who emphasize the need to balance ecological, ethical, and biosecurity considerations. Education on the proper handling of wild boar carcasses and encouraging the reporting of suspected cases is regularly cited as one of the most effective prevention methods [22]. Experiences from the Baltic countries show that hunters who participate in workshops and training sessions have significantly greater confidence in their own abilities and are more willing to actively cooperate with veterinary services [7,44]. Such activities also strengthen the sense of responsibility and shared mission among hunters, which is crucial for effectively controlling the spread of ASF. Another recurring challenge identified in European studies is the lack of investment in disease control infrastructure. In Germany and Latvia, hunters often emphasize the need for financial support from the government for implementing preventive measures, particularly regarding the costs of PCR testing and potential vaccination of wild boar [7,24].

The results of this study are consistent with the previously mentioned studies and clearly show that hunters’ attitudes towards ASF control are grouped around three interrelated areas: communication and awareness, institutional coordination and regulatory measures. These patterns have direct policy relevance. First, the finding that middle-aged hunters (35–54 years) expressed significantly higher support for institutional capacity and coordination suggests that this group should be formally deployed as facilitators within district-level task forces, where they can translate institutional priorities into practise and mobilise younger hunters. Second, the observation that hunters concerned about ASF showed stronger support for regulatory measures suggests that communication strategies should not remain generic but should explicitly demonstrate the effectiveness of specific measures such as carcass removal protocols or border controls in reducing the spread of disease. Finally, the overwhelming expectation (96.0%) that the government should fund vaccination and testing emphasises the need for transparent compensation and funding systems that can contribute to greater hunter engagement and more consistent implementation of biosecurity measures. Without such mechanisms, there is a risk that stated support for ASF interventions will be undermined by practical or financial constraints. By operationalising these evidence-based findings, authorities can move beyond rhetorical appeals for cooperation with hunters and instead create concrete, permanent structures for participatory disease management.

Overall, the literature emphasizes that establishing effective cooperation between hunters, veterinary services, and livestock producers is crucial for controlling ASF [5]. In this regard, Croatia can draw on the experiences of countries that have already developed successful protocols, such as Latvia, where integrated training programs and financial incentives have led to improved control over the spread of the disease [7].

## 5. Conclusions

Before drawing our conclusions, we should mention that, to our knowledge, this is the first study in Croatia to systematically investigate the self-reported knowledge, attitudes and behaviour of wild boar hunters in relation to African swine fever (ASF), thus providing a unique evidence base for future policy and management strategies. This novelty is offset by certain limitations: the relatively small sample size, the use of an online survey and the predominance of older wild boar hunters, people with secondary education and rural dwellers in the group of respondents could limit the representativeness of the results. In addition, the use of non-probability sampling limits generalizability; findings may reflect engaged hunters rather than the broader community. While the online format allowed for cost-effective data collection across a large geographic area with a limited budget, it also carries the risk of sample bias especially for populations with unequal access to digital tools or varying levels of technological literacy. These limitations should be taken into account when interpreting the results. However, they do not diminish the importance of the results, which can directly inform targeted education, communication, and cooperation strategies between wild boar hunters, veterinary authorities, and policy makers. Although this study focussed on a specific Croatian county recently affected by ASF, the results obtained are also relevant for other regions facing similar challenges, especially in Central and Eastern Europe with comparable hunting cultures.

This study underscores the crucial role of wild boar hunters in African swine fever (ASF) prevention and control in Croatia. To translate the findings into practical guidance for policy and management, several key conclusions and recommendations can be drawn:

- Hunters should be recognised as active contributors to biosecurity, especially in early detection, reporting, and preventive measures.
- Effective ASF control requires clear, reliable, and two-way communication between hunters, veterinary authorities, and policy makers.
- Education and awareness-raising are essential. Training needs to combine traditional workshops with modern digital tools to reach the different groups of hunters.
- ASF management should take a cross-sectoral approach, combining social and cultural dimensions with veterinary and environmental strategies.
- Policy makers should involve hunters in decision-making processes and ensure transparency to build trust and compliance.
- Hunters must be seen as strategic partners in integrated wildlife health management. Future research should focus on mechanisms to engage and evaluate education and cooperation initiatives.

## Figures and Tables

**Table 1:** Descriptive statistics of the socio-demographic and hunting characteristics of the respondents.

		*N*	(%)
Gender	male	259	93.8
	female	17	6.2
Age	younger respondents (18–34)	123	44.5
	middle-aged respondents (35–54)	131	47.5
	older respondents (55+)	22	8.0
Education	secondary education or lower	185	67.0
	college degree or higher	91	33.0
Hunting experience	<5 years	135	48.9
	>5 years	141	51.1
Hunt	professional hunting	20	7.2
	recreational hunting	256	92.8
Hunting during the year	once a month or less	72	26.1
	very often	204	73.9
	group hunting with dogs	123	44.6
Preferred hunting method	individual hunting	153	55.4
Member of hunting associations	yes	225	81.5
	no	51	18.5
Attendance of hunting seminars or trainings	never to rarely	162	58.7
	often to regularly	114	41.3
Familiarity with the term ASF	fully familiar	258	93.5
	little or not familiar	18	6.5
Main source of information about ASF	television	51	18.5
	radio	2	0.7
	newspapers/print media	19	6.9
	internet (news portals, social media)	183	66.3
	other	21	7.6
Concern about the spread of the disease	concerned	201	72.8
	not concerned	75	27.2
Confidence in recognizing the disease	not confident	122	44.2
	confident	154	55.8
Measures to prevent disease spread *	zoning and movement restrictions	81	29.3
	PCR testing of dead wild boars	115	41.7
	PCR testing of shot wild boars	132	47.8
	installation of physical barriers	25	9.1
	depopulation	89	32.2
	selective hunting	85	30.8
	vaccination of wild boars	76	27.5
Who should finance wild boar vaccination	government	265	96.0
	pig producers	11	4.0

Note: * Number of respondents do not match 276 because several options could be selected.

**Table 2:** Hunters’ level of agreement with various statements related to ASF control, education, cooperation and prevention.

	Me	Mo	M	SD	%
Preventive measures					
I believe surveillance of wild boars should be intensified in areas where ASF is present.	4	5	3.99	1.172	76.8
I believe preventive measures are key in controlling the spread of ASF.	4	5	3.86	1.215	67.1
I believe stricter border controls are needed to prevent ASF introduction.	4	5	3.82	1.345	66.7
I think stricter legal measures are needed to prevent ASF from entering new areas.	4	5	3.49	1.309	53.6
Education and awareness about ASF					
Hunters should be educated on proper disposal and destruction of wild boar remains to prevent disease spread.	5	5	4.33	1.011	87.7
Hunters should be encouraged to report suspected ASF cases.	5	5	4.33	0.986	89.8
Hunters should be educated about ASF symptoms.	5	5	4.32	0.984	88.4
Information about ASF should be regularly updated and monitored.	5	5	4.26	1.026	86.5
I believe it is important to educate hunters on proper handling of wild boars to prevent disease spread.	5	5	4.22	1.075	85.1
I believe the local community should be educated about the risks associated with ASF.	4	5	4.21	1.044	86.2
Awareness of the importance of vaccinating domestic pigs against ASF should be raised.	4	5	4.16	1.094	82.6
Hunters should regularly check wild boars for signs of ASF.	4	5	4.07	1.086	78.6
Cooperation and support					
I believe adequate support should be provided to hunters in combating ASF.	5	5	4.49	0.912	92.4
I believe hunters should be encouraged to cooperate and share information about suspected ASF cases.	5	5	4.31	0.960	90.2
I believe international cooperation is crucial in controlling ASF.	5	5	4.31	1.018	87.3
I believe cooperation between hunters and local authorities is essential in the fight against ASF.	5	5	4.29	1.032	84.1
Limited resources and the need for investment					
I think more investment is needed in infrastructure for ASF control and prevention.	4	5	4.18	0.988	82.9
I believe national programs should be established for the monitoring and continuous control of ASF.	4	5	4.18	0.982	84.7
I believe more experts should be engaged in ASF control.	4	5	4.15	0.989	81.1

Notes: Me = median; Mo = mode; M = arithmetic mean; SD = standard deviation; % = agree/strongly agree.

**Table 3:** Exploratory factor analysis of hunters’ attitudes toward ASF prevention, education, cooperation, and resource constraints.

	F1–ASF Communication, Education, and Awareness	F2–Institutional Capacity and Coordination	F3–Regulation and Disease Control Policy
Information about ASF should be regularly updated and monitored.	**0.825**	0.345	0.255
I believe hunters should be encouraged to cooperate and share information about suspected ASF cases.	**0.812**	0.414	0.245
Hunters should be educated on proper disposal and destruction of wild boar remains to prevent disease spread.	**0.799**	0.309	0.256
Hunters should be encouraged to report suspected ASF cases.	**0.794**	0.367	0.222
Awareness of the importance of vaccinating domestic pigs against ASF should be raised.	**0.781**	0.191	0.262
I believe the local community should be educated about the risks associated with ASF.	**0.781**	0.382	0.247
Hunters should regularly check wild boars for signs of ASF.	**0.779**	0.253	0.183
I believe it is important to educate hunters on proper handling of wild boars to prevent disease spread.	**0.76**	0.378	0.212
Hunters should be educated about ASF symptoms.	**0.736**	0.404	0.243
I believe more experts should be engaged in ASF control.	0.220	**0.799**	0.235
I believe national programs should be established for the monitoring and continuous control of ASF.	0.272	**0.785**	0.208
I believe international cooperation is crucial in controlling ASF.	0.377	**0.78**	0.128
I believe cooperation between hunters and local authorities is essential in the fight against ASF.	0.337	**0.764**	0.145
I believe adequate support should be provided to hunters in combating ASF.	0.491	**0.698**	0.111
I think more investment is needed in infrastructure for ASF control and prevention.	0.413	**0.666**	0.273
I think stricter legal measures are needed to prevent ASF from entering new areas.	0.082	0.183	**0.822**
I believe surveillance of wild boars should be intensified in areas where ASF is present.	0.357	0.130	**0.774**
I believe stricter border controls are needed to prevent ASF introduction.	0.220	0.178	**0.765**
I believe preventive measures are key in controlling the spread of ASF.	0.330	0.195	**0.674**
% Variance Explained	34.922	24.050	16.020
Eigenvalues	6.635	4.570	3.044
Cronbach’s Alpha	0.966	0.922	0.834

KMO = 0.945, Bartlett’s χ^2^ = 5153.191, *p* = 0.000; Extraction Method: Principal Component Analysis. Rotation Method: Varimax with Kaiser Normalization; Bold numbers are used to emphasise the highest loading of each statement

## Data Availability

The data obtained in the survey can be retrieved from the corresponding author upon reasonable request.
